# A CLIENT-CENTERED HOME TRAINING APP OF MUSIC-BASED THERAPY FOR UPPER EXTREMITY FUNCTIONS IN OLDER ADULTS WITH STROKE

**DOI:** 10.1093/geroni/igae098.3774

**Published:** 2024-12-31

**Authors:** Yi-An Chen, Sara Lynn Johnstone, Annie Solomon, Martin Norgaard, Gil Weinberg, Dhruv Pargai, Evan Szypulski, Jon Sanford

**Affiliations:** Georgia State University, Atlanta, Georgia, United States; Georgia State University, Atlanta, Georgia, United States; Georgia State University, Atlanta, Georgia, United States; Georgia State University, Atlanta, Georgia, United States; Georgia Institute of Technology, Atlanta, Georgia, United States; Georgia Institute of Technology, Atlanta, Georgia, United States; Georgia State University, Atlanta, Georgia, United States; Georgia State University, Atlanta, Georgia, United States

## Abstract

Music-based therapy (MBT) has been shown to be effective in improving upper extremity (UE) functions for older adults with stroke in clinical settings. To expand the MBT benefits to home, we designed a client-centered, stroke-specific MBT app and examined the app feasibility with stroke survivors in this study. During the 2-week pilot home testing, the participants (n=5, mild to moderate UE motor impairments) actively used the app for rehab exercises around 71% of the time, which equals 26.6 ± 16.7 min per day, 5 days per week, over 2 weeks. Motor outcomes portrayed a positive trend from pre- to post-intervention. Participants’ average Fugl-Meyer Assessment scores increased from 53 to 57, and Nine-Hole Pegboard Test time decreased from 38 seconds to 32 seconds. Additionally, participants provided generally positive feedback, commenting on the app user-friendliness (e.g., easy to navigate, clear exercise instructions, convenience) and motivational music feature (e.g., engaging and a new way to experience therapy). They also enjoyed the flexibility of training scheduling – participants could do rehab exercises at any time they wanted. Based on the positive findings, we anticipate that the app will serve as a feasible tool for older adults with stroke to engage in a personalized program to improve UE functions at home. This will minimize the burden of travel and wait times to healthcare clinics and increase rehab access for older adults. We are currently continuing the data collection and app refinement to optimize the app to support stroke recovery in older adults.

